# The level of cognitive function and recognition of emotions in older adults

**DOI:** 10.1371/journal.pone.0185513

**Published:** 2017-10-04

**Authors:** Marianna Virtanen, Archana Singh-Manoux, G. David Batty, Klaus P. Ebmeier, Markus Jokela, Catherine J. Harmer, Mika Kivimäki

**Affiliations:** 1 Finnish Institute of Occupational Health, Helsinki, Finland; 2 Department of Epidemiology and Public Health, University College London, London, United Kingdom; 3 Inserm, Centre for Research in Epidemiology and Population Health, Villejuif, France; 4 Centre for Cognitive Ageing & Cognitive Epidemiology, University of Edinburgh, Edinburgh, United Kingdom; 5 Department of Psychiatry, University of Oxford, Warneford Hospital, Oxford, United Kingdom; 6 Department of Psychology and Logopedics, University of Helsinki, Helsinki, Finland; 7 Clinicum, Faculty of Medicine, University of Helsinki, Helsinki, Finland; University of Groningen, NETHERLANDS

## Abstract

**Background:**

The association between cognitive decline and the ability to recognise emotions in interpersonal communication is not well understood. We aimed to investigate the association between cognitive function and the ability to recognise emotions in other people’s facial expressions across the full continuum of cognitive capacity.

**Methods:**

Cross-sectional analysis of 4039 participants (3016 men, 1023 women aged 59 to 82 years) in the Whitehall II study. Cognitive function was assessed using a 30-item Mini-Mental State Examination (MMSE), further classified into 8 groups: 30, 29, 28, 27, 26, 25, 24, and <24 (possible dementia) MMSE points. The Facial Expression Recognition Task (FERT) was used to examine recognition of anger, fear, disgust, sadness, and happiness.

**Results:**

The multivariable adjusted difference in the percentage of accurate recognition between the highest and lowest MMSE group was 14.9 (95%CI, 11.1–18.7) for anger, 15.5 (11.9–19.2) for fear, 18.5 (15.2–21.8) for disgust, 11.6 (7.3–16.0) for sadness, and 6.3 (3.1–9.4) for happiness. However, recognition of several emotions was reduced already after 1 to 2-point reduction in MMSE and with further points down in MMSE, the recognition worsened at an accelerated rate.

**Conclusions:**

The ability to recognize emotion in facial expressions is affected at an early stage of cognitive impairment and might decline at an accelerated rate with the deterioration of cognitive function. Accurate recognition of happiness seems to be less affected by a severe decline in cognitive performance than recognition of negatively valued emotions.

## Introduction

The ability to recognize emotions is a key element in maintaining interpersonal relationships in aging and an important resource for mental health [1]. Newborns and infants younger than 5 months have the preference of smiling faces, which has been suggested to be automatic and universal and reflect their earliest experience of human face [2, 3]. Later in life, deficiency in emotion recognition has been associated with withdrawal from social interaction, poor communication, and mental health problems [4–6]. In clinical studies, poorer recognition of emotions in facial expressions has also been linked to Alzheimer’s disease [4, 7, 8] in which interpersonal problems have been identified as important targets for intervention [9].

Cognitive function, such as short-term memory, speed of reasoning and reaction time, tend to decrease at older ages even in the absence of neurodegenerative disorders, such as Alzheimer’s disease. With population ageing, ‘cognitive ageing’ has become a major public health issue affecting every day functioning of an increasing proportion of people [10, 11]. Tests of emotion recognition might represent a potential tool for detection of early-stage cognitive impairment. In addition, research on potential mechanisms, such as deficits in the recognition of other people’s emotions, which might link impaired cognitive function to interpersonal problems and behavioural disturbances, would increase our understanding of life with cognitive impairment in old age.

However, while severe states of neurodegenerative diseases have been shown to be associated with impaired recognition of emotion, knowledge of the degree to which ‘mild cognitive impairment’ is associated with impaired recognition of emotions, is limited. Studies that have included participants with mild cognitive impairment have been small in scale and have produced mixed findings [12–18]. Analyses of more than 4000 participants allowed us to more reliably examine the link between cognitive function and recognition of facial expressions across the full continuum of cognitive capacity, ranging from minor cognitive impairment to possible dementia.

## Methods

### Ethics statement

Ethical approval for the Whitehall II study was obtained from the University College London Medical School and the NHS London-Harrow Health Research Authority committees on the ethics of human research; all participants provided written informed consent. All data were analyzed anonymously.

### Participants and study design

The Whitehall II study is a prospective cohort study of British civil servants established to identify social determinants of health [19]. The initial study population was all London-based office staff, aged 35–55 years, working in 20 departments on recruitment to the study in 1985–8. With a response proportion of 73%, the initial cohort consisted of 10308 participants who have now been followed up for over 25 years. In the present analyses we used cross-sectional data from the 2012–2013 clinical examination when the Facial Expression Recognition Task (FERT) was administered for the first time. A total of 6318 individuals participated in that phase, of whom 4505 (71.3%) agreed to participate in testing using the FERT. After the exclusion of 43 participants with missing data on Mini-Mental State Examination (MMSE) or important covariates (n = 423) [20], i.e., educational level, depressive symptoms, or antidepressant use, the analytical sample of 4039 participants included 3016 (74.7%) men and 1023 (25.3%) women aged 59 to 82.

After complete description of the study to the participants, all participants provided written informed consent. Ethical approval for the Whitehall II study was obtained from the University College London Medical School and the NHS London-Harrow Health Research Authority committees on the ethics of human research. Whitehall II data, protocols, and other metadata are available to bona fide researchers for research purposes (see the Whitehall II data sharing policy at http://www.ucl.ac.uk/whitehallII/data-sharing).

### Cognitive function

We used the 30-item version of the Mini-Mental State Examination (MMSE) adapted to the United Kingdom, to assess cognitive function [21, 22]. The MMSE is a common method of screening for dementia, and correlates well with a number of cognitive screening scores and neuropsychological tests [23, 24]. To differentiate ‘possible dementia’ or clinical cognitive impairment from non-clinical cognitive states, a conventional cut-point for <24 was used [24, 25], resulting in the following categories of cognitive function: 30 points (reference), 29, 28, 27, 26, 25, 24, and <24. We used four MMSE subscores (attention/concentration, language skills, memory recall and orientation) for a sensitivity analysis.

### Recognition of emotion in facial expressions

The FERT, featuring neutral and five basic facially-depicted emotions: anger, fear, disgust, sadness, and happiness, was derived from 10 individual characters in the Pictures of Facial Affect Series [26], which has been morphed between each prototype and neutral [27]. The procedure involves taking a variable percentage (10% increments) of the shape and texture differences between two standard images which ranged from 0% (neutral) to 100% (full emotion). We used a computerized version in which three examples of each emotion at each intensity were presented (5 emotions × 10 intensities × 3 examples = 150 stimuli) [28]. With each face was also depicted in a neutral expression, a total of 160 stimuli were presented. The stimuli were presented in random order on a computer screen for 500 milliseconds after which they were replaced by a blank screen. The FERT test includes adult, Caucasian and both male and female faces. Participants were asked to respond as quickly and as accurately as possible by pressing one of six labelled keys although the test was not time-limited. Accuracy was calculated as a percentage of correct identification out of 30 expressions in each emotion (out of 10 in the neutral expression). Misclassification, that is, bias towards any of the emotions measured, was assessed by calculating the percentage of false recognition out of the 130 expressions (out of 150 in the neutral expression); for example, the percentage of recognizing fear in incorrect responses to 130 expressions other than fear.

### Covariates

Age, sex and self-reported years of education, which was requested at phase 5 survey (1997–9) and was categorized as <13, 13–15, 16–18, >18 years were sociodemographic factors examined in this study and were chosen because they may confound the association between cognitive function and recognition of emotions. Furthermore, the Clinical Interview Schedule-Revised (CIS-R) [29] was used to identify participants with depressive symptoms. CIS-R is a structured diagnostic interview from symptom scores for 14 psychiatric symptoms can be generated. A self-administered computerized version of CIS-R was used in this study [30], including subscores of depression (anhedonia, sadness, feeling miserable, loss of interest in things, and feeling guilty without a clear reason) and depressive ideas (feeling worthless, feeling hopeless, feeling that life is not worth living, and suicidal thoughts). ‘Caseness’ was identified as a score ≥2 in either of the sub-scores. Antidepressant use (yes/no) was based on the survey questionnaire [28].

### Statistical analyses

We used multivariable analysis of variance to calculate difference in the mean scores and 95% confidence interval between the MMSE score groups for each FERT subtest, with the highest MMSE score (30 points) as the reference group. The first model was adjusted for age and the second model was adjusted for age, sex, educational level, depressive symptoms and antidepressant use. We tested curvilinear trend in separate analyses by adding the squared continuous MMSE score variable to the models in addition to the main effect of MMSE. Significant curvilinear trend was indicated if the MMSE squared term produced significant association with FERT outcomes in the model. As a sensitivity analysis, we examined the associations using FERT misclassification rate as the outcome. Bonferroni correction to *P*-values (i.e., multiplying *P*-value with the number of comparisons, 7) was used to control for multiple testing in an additional sensitivity analysis. Finally, we calculated bivariate Pearson correlation between MMSE subscores and FERT accuracy test scores for each emotion. All analyses were performed with SAS version 9.4 (SAS Institute Inc., Cary, NC, USA).

## Results

The mean age of participants was 69.1 years, 25.3% were women, and 25.4% had less than 13 years of education (Table 1). Depressive symptoms were reported by 7.3% and antidepressant use by 4.1%. Sixty-seven participants (1.7%) had the MMSE-score of <24 (range 7 to 23; 18 participants had less than 21 points) and 841 (20.8%) had the highest score (30 points). In descending order, the highest accuracy in recognition of emotions was found for happiness (mean of 69.3% correct), followed by ‘neutral’ (66.1%), fear (57.2%), disgust (54.3%), anger (50.8%), and sadness (45.8%).

**Table 1:** Characteristics of the Whitehall II study participants (n = 4039).

Characteristic	Mean (SD) or N (%)
Age, Mean (SD)	69.1 (5.6)
Sex, N (%)	
Men	3016 (74.7)
Women	1023 (25.3)
Years in education, N (%)	
<13	1027 (25.4)
13–15	981 (24.3)
16–18	1250 (31.0)
>18	781 (19.3)
Depressive symptoms, N (%)	
No	3744 (92.7)
Yes	295 (7.3)
Antidepressant use, N (%)	
No	3874 (95.9)
Yes	165 (4.1)
Mini Mental State Examination (MMSE) score, N (%)	
<24 (possible dementia)	67 (1.7)
24	59 (1.5)
25	103 (2.6)
26	255 (6.3)
27	529 (13.1)
28	936 (23.2)
29	1249 (30.9)
30	841 (20.8)
Facial Expression Recognition Task (FERT) score	
Accuracy, Mean (SD)	
Anger	50.8 (15.6)
Fear	57.2 (15.2)
Disgust	54.3 (13.6)
Sadness	45.8 (17.8)
Happiness	69.3 (12.6)
Neutral	66.1 (19.6)
Misclassification, Mean (SD)	
Anger	3.9 (3.5)
Fear	5.9 (4.2)
Disgust	4.5 (4.0)
Sadness	5.6 (4.4)
Happiness	3.1 (3.6)
Neutral	26.5 (8.8)

**Fig 1:**
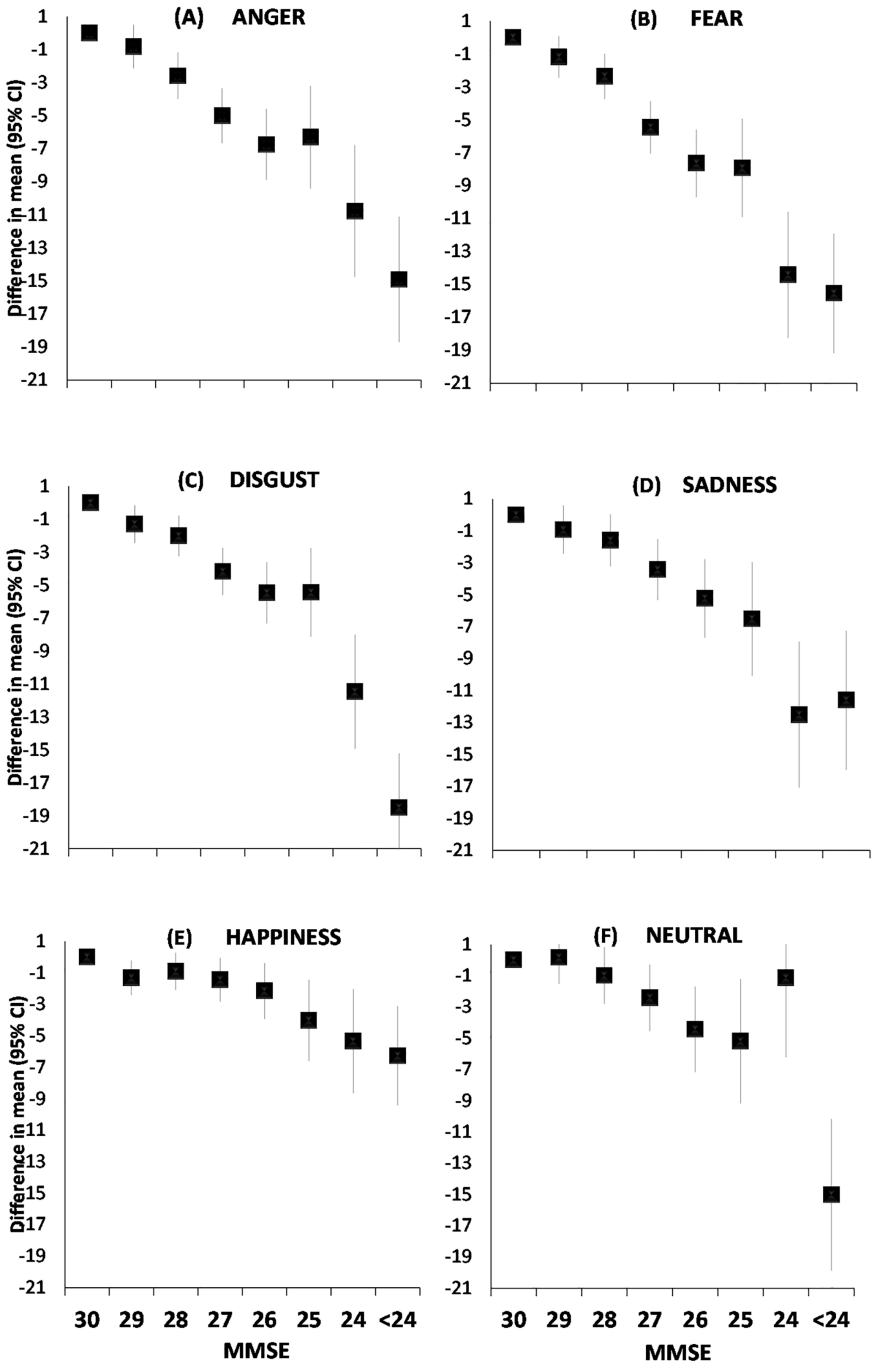
(panels A to F) Multivariable adjusted association between the Mini-Mental State Examination (MMSE) cognitive function test score and accuracy of emotion recognition in the Facial Expression Recognition Task (FERT)^a^. ^a^Point estimates are mean and error bars 95% confidence intervals. Models are adjusted for age, sex, educational level, depressive symptoms, and antidepressant use.

There were strong associations between the total MMSE cognitive function score and all accuracy task outcomes in the age, sex, education, depressive symptoms and antidepressant use adjusted models (Fig 1, panels A to F, S1 Table, Models 2). In general, study members with greater cognitive impairment had lower accuracy across all FERT accuracy domains and decreased performance in accuracy was evident already at minor (1 to 3-point) deficits in cognitive performance. The Bonferroni-corrected analyses did not remarkably change this picture; Compared to the highest MMSE group (score = 30), the first significant decline in emotion recognition was observed at the MMSE score of 28 in accuracy of anger, fear, and disgust, 27 for sadness, and 25 for happiness.

The association was curvilinear in all accuracy tests (*p*≤ 0.023 for curvilinear trend), except for anger and happiness for which the association was linear (*p*< 0.0001 for linear term). In this case, curvilinearity suggests that facial expression recognition deteriorated with an accelerated rate along with lowering cognitive function scores. There was some variation in the difference between mean accuracy in the recognition of different emotions: the multivariable adjusted difference between the highest MMSE group and the group with the lowest score was 14.9 for anger, 15.5 for fear, 18.5 for disgust, 11.6 for sadness, 6.3 for happiness and 15.0 for neutral (S1 Table). Thus, recognition of happiness seemed to be less affected by a severe decline in cognitive function than other emotions.

The findings using an alternative indicator for emotion recognition, that is, emotion misclassification, largely replicated the findings on accuracy (Fig 1; S2 Table). The associations were curvilinear (all *p*≤ 0.016 for curvilinear trend), except for misclassification of neutral emotion where the trend was linear (*p* = 0.020). Multivariable adjusted mean difference in misclassification was 3.3 for anger, 4.0 for fear, 4.7 for disgust, 4.0 for sadness, 3.4 for happiness and -3.5 for neutral (S2 Table). Bonferroni-corrected analyses did not change the main conclusions.

S3 Table shows bivariate correlations between MMSE total score and subscores and accuracy of emotion recognition in the FERT, suggesting a slightly stronger association of the total MMSE score with recognition of emotions than any of the MMSE subscores. Thus, it seems that the findings are based on the global cognitive capacity rather than a deficit in any particular component of cognitive function.

## Discussion

In this study among 4039 older adults, we examined the association between cognitive function and recognition of emotions in facial expressions across the full continuum of cognitive capacity. The main finding in our study was that the ability to recognize emotion in facial expressions might be affected at a relatively early phase of cognitive decline. Furthermore, a curvilinear association that we observed in several tests suggests that the capacity to recognize emotions may decline at an accelerated rate with the deterioration of cognitive function. The findings were robust to adjustment for age, sex, educational level, depressive symptoms and antidepressant use.

This evidence extends research on this field by showing that cognitive decline might not only relate to memory, executive function and reaction time, but also to processing of emotional information. Our findings add to the evidence obtained from earlier smaller-scale clinical studies showing that disturbance in various neurocognitive processes (e.g. vigilance, speed of processing, and cognitive flexibility) decreases a person’s ability to recognize emotion in facial expressions [31–35]. Impaired recognition of emotions has been found among patients with Alzheimer’s disease [4, 7, 8] while findings among participants with mild cognitive impairment have been mixed [12–18].

We demonstrated that depending on emotion, the performance in the accuracy test started to decline from 27 to 29 MMSE points out of the maximum 30 points. Scores 23 or less are used to define ‘possible dementia’ whereas the scores between 24 and 30 are within the normal range of cognitive function, although the low-bound scores might be associated with minor cognitive impairment [24, 25]. The few previous experimental studies available on this issue are small, used varying definitions of ‘mild cognitive impairment’ [14–16]. Although mild and moderate Alzheimer’s disease have been found to adversely affect components of attention [36], these deficits may be present already before the manifest disease and may further reduce the performance in facial expression recognition.

Not all emotions seem to be affected to the same extent by cognitive function. The adjusted mean difference in accuracy between the best and worst performed MMSE groups was 11.6 to 18.5 in other emotions but only 6.3 for happiness, suggesting that recognition of emotions with negative valence might be more strongly affected than recognition of happiness. Interestingly, infants younger than 5 months prefer happy faces over other emotions [2, 3]; we might hypothesise that happy emotion is also the last one to be affected by severe cognitive decline although this observations needs confirmation in other studies.

Clinical studies suggest better recognition of negative than positive stimuli among patients with Alzheimer’s disease [37]. However, the present cohort was from a general population and capable of performing the tests, thus, it is unlikely that many of the participants in our study were at an advanced stage of Alzheimer’s disease or dementia. A future line of research could apply neuroimaging studies to analyse the potential relationship of recognition of facial emotions with accelerated cognitive ageing (not associated with a neurodegenerative disease), Alzheimer’s disease, other dementias, and small vessel cerebral ischemia which has been found to be associated with mild cognitive impairment [38–40]. It would be important to examine whether different types of stimuli, e.g., static versus dynamic, produce different findings [41]. It is also important to note that poor performance on emotion recognition tasks might reflect impaired cognitive function rather than impaired emotion processing, per se, for example, with difficulties in following task instructions or technical performance of tasks [14]. However, neuroimaging data supports the hypothesis that impaired emotion recognition might indicate early signs of impaired cognitive processing [39].

Two leading theories to explain emotion recognition are Theory-Theory and Simulation Theory [42]. Theory-Theory emphasises rule-based cognitive processing in interpretation of other people’s mental states, involving the use of conceptual (semantic) emotion-related knowledge [43]. In Simulation Theory, the emphasis is on the other cognitive processes rather than appraisal, such as motivational and physiological changes and the subjective emotional experiences [44]. Simulation Theory postulates that humans recognize emotions by simulating the other people’s emotional state in themselves. Although within the present study, we did not directly test these theories, they highlight complex cognitive processes involved in emotion recognition, which helps us to understand the findings.

A strength of the present study is a detailed analysis of the large range of cognitive performance which allowed us to show that recognition of emotions in facial expressions starts to decline already at the an early phase of impaired cognitive performance. Also a novel finding in our study was a curvilinear trend between the MMSE scores and recognition of several emotions; that is, the association between poorer cognitive function and poorer recognition of facial expressions accelerated along with the deterioration of cognitive function.

Limitations of this study should be noted. The cross-sectional study design prevents us from making inferences about the direction of the associations. Furthermore, the MMSE has been developed to provide a brief screening test that quantitatively assesses the level of cognitive impairment and cognitive changes over time, for example, among patient groups [23]. Thus, the MMSE does not provide a diagnostic tool to detect dementia which, however was not the focus of our study. Moreover, as negative emotions elicit a higher arousal level than the positive ones, arousal level might act as a mediator or effect modifier which should be examined in future studies. We had only one emotion with positive valence (i.e., happiness); the rest of the emotions had negative valence [1]. In the future, it would be important to examine whether equal number of emotions with negative and positive valence in the study would lead to similar findings with ours. Although an advantage of our study population was its large size, it represents an elderly, originally occupational cohort and therefore the findings may not be generalizable to the total population of the United Kingdom.

Keeping in mind these limitations, this study provides new evidence on the association between cognitive function and recognition of emotion in facial expressions. Because recognition of emotions in other people’s faces is a central aspect of social interaction, our findings add to understanding of the determinants of interpersonal problems and mental health in older people.

## Supporting information

S1 Table Association between the Mini-Mental State Examination (MMSE) cognitive function test score and accuracy of emotion recognition in the Facial Expression Recognition Task (FERT). (DOCX)

S2 Table Association between the Mini-Mental State Examination (MMSE) cognitive function test score and misclassification of emotion recognition in the Facial Expression Recognition Task (FERT). (DOCX)

S3 Table Bivariate Pearson correlations (r) between Mini Mental State Examination (MMSE) total score and subscores and accuracy of emotion recognition in the Facial Expression Recognition Task (FERT). (DOCX)

## References

[1] Ekman P, Oster H. Facial expressions of emotions. Annu Rev Psychol. 1979;30:527–54.

[2] Leppanen JM, Nelson CA. Tuning the developing brain to social signals of emotions. Nat Rev Neurosci. 2009;10:37–47. doi: 10.1038/nrn2554 . 19050711 10.1038/nrn2554 PMC2976651

[3] Bayet L, Quinn PC, Tanaka JW, Lee K, Gentaz E, Pascalis O. Face gender influences the looking preference for smiling expressions in 3.5-month-old human infants. PLoS One. 2015;10:e0129812 doi: 10.1371/journal.pone.0129812 . 26068460 10.1371/journal.pone.0129812 PMC4465895

[4] Elamin M, Pender N, Hardiman O, Abrahams S. Social cognition in neurodegenerative disorders: a systematic review. J Neurol Neurosurg Psychiatry. 2012;83:1071–9. doi: 10.1136/jnnp-2012-302817 . 22869923 10.1136/jnnp-2012-302817

[5] Ruffman T, Henry JD, Livingstone V, Phillips LH. A meta-analytic review of emotion recognition and aging: implications for neuropsychological models of aging. Neurosci Biobehav Rev. 2008;32:863–81. doi: 10.1016/j.neubiorev.2008.01.001 . 18276008 10.1016/j.neubiorev.2008.01.001

[6] Szanto K, Dombrovski AY, Sahakian BJ, Mulsant BH, Houck PR, Reynolds CF 3rd, et al Social emotion recognition, social functioning, and attempted suicide in late-life depression. Am J Geriatr Psychiatry. 2012;20:257–65. doi: 10.1097/JGP.0b013e31820eea0c . 22354116 10.1097/JGP.0b013e31820eea0c PMC3286029

[7] Klein-Koerkamp Y, Beaudoin M, Baciu M, Hot P. Emotional decoding abilities in Alzheimer's disease: a meta-analysis. J Alzheim Dis. 2012;32:109–25. doi: 10.3233/JAD-2012-120553 . 22776967 10.3233/JAD-2012-120553

[8] Kumfor F, Irish M, Leyton C, Miller L, Lah S, Devenney E, et al Tracking the progression of social cognition in neurodegenerative disorders. J Neurol Neurosurg Psychiatry. 2014;85:1076–83. doi: 10.1136/jnnp-2013-307098 . 24569686 10.1136/jnnp-2013-307098

[9] Spector A, Charlesworth G, King M, Lattimer M, Sadek S, Marston L, et al Cognitive-behavioural therapy for anxiety in dementia: pilot randomised controlled trial. Br J Psychiatry. 2015;206:509–16. doi: 10.1192/bjp.bp.113.140087 . 25698766 10.1192/bjp.bp.113.140087

[10] Blazer DG, Yaffe K, Karlawish J. Cognitive aging: a report from the Institute of Medicine. JAMA. 2015;313:2121–2. doi: 10.1001/jama.2015.4380 . 25875498 10.1001/jama.2015.4380

[11] Rajan KB, Hebert LE, Scherr P, Dong X, Wilson RS, Evans DA, et al Cognitive and physical functions as determinants of delayed age at onset and progression of disability. J Gerontol A Biol Sci Med Sci. 2012;67:1419–26. doi: 10.1093/gerona/gls098 . 22539654 10.1093/gerona/gls098 PMC3636674

[12] Lee SB, Koo SJ, Song YY, Lee MK, Jeong YJ, Kwon C, et al Theory of mind as a mediator of reasoning and facial emotion recognition: findings from 200 healthy people. Psychiatry Inv. 2014;11:105–11. doi: 10.4306/pi.2014.11.2.105 . 24843363 10.4306/pi.2014.11.2.105 PMC4023082

[13] Horning SM, Cornwell RE, Davis HP. The recognition of facial expressions: an investigation of the influence of age and cognition. Neuropsychol Dev Cognit Sect B. 2012;19:657–76. doi: 10.1080/13825585.2011.645011 . 22372982 10.1080/13825585.2011.645011

[14] McCade D, Savage G, Naismith SL. Review of emotion recognition in mild cognitive impairment. Dementia Geriatr Cognit Disord. 2011;32:257–66. doi: 10.1159/000335009 . 22222774 10.1159/000335009

[15] Henry JD, Thompson C, Rendell PG, Phillips LH, Carbert J, Sachdev P, et al Perception of biological motion and emotion in mild cognitive impairment and dementia. J Int Neuropsychol Soc. 2012;18:866–73. doi: 10.1017/S1355617712000665 . 22687579 10.1017/S1355617712000665

[16] Varjassyova A, Horinek D, Andel R, Amlerova J, Laczo J, Sheardova K, et al Recognition of facial emotional expression in amnestic mild cognitive impairment. J Alzheim Dis. 2013;33:273–80. doi: 10.3233/JAD-2012-120148 . 22954669 10.3233/JAD-2012-120148 PMC3918473

[17] Weiss EM, Kohler CG, Vonbank J, Stadelmann E, Kemmler G, Hinterhuber H, et al Impairment in emotion recognition abilities in patients with mild cognitive impairment, early and moderate Alzheimer disease compared with healthy comparison subjects. Am J Geriatr Psychiatry. 2008;16:974–80. doi: 10.1097/JGP.0b013e318186bd53 . 19038896 10.1097/JGP.0b013e318186bd53

[18] Spoletini I, Marra C, Di Iulio F, Gianni W, Sancesario G, Giubilei F, et al Facial emotion recognition deficit in amnestic mild cognitive impairment and Alzheimer disease. Am J Geriatr Psychiatry. 2008;16:389–98. doi: 10.1097/JGP.0b013e318165dbce . 18403572 10.1097/JGP.0b013e318165dbce

[19] Marmot M, Brunner E. Cohort Profile: the Whitehall II study. Int J Epidemiol. 2005;34:251–6. doi: 10.1093/ije/dyh372 . 15576467 10.1093/ije/dyh372

[20] Harmer CJ, Cowen PJ. 'It's the way that you look at it'—a cognitive neuropsychological account of SSRI action in depression. Phil Transact R Soc Lond Ser B. 2013;368:20120407 doi: 10.1098/rstb.2012.0407 . 23440467 10.1098/rstb.2012.0407 PMC3638386

[21] Folstein MF, Folstein SE, McHugh PR. "Mini-mental state". A practical method for grading the cognitive state of patients for the clinician. J Psychiatr Res. 1975;12:189–98. . 1202204 10.1016/0022-3956(75)90026-6

[22] Singh-Manoux A, Dugravot A, Brunner E, Kumari M, Shipley M, Elbaz A, et al Interleukin-6 and C-reactive protein as predictors of cognitive decline in late midlife. Neurology. 2014;83:486–93. doi: 10.1212/WNL.0000000000000665 . 24991031 10.1212/WNL.0000000000000665 PMC4141998

[23] Tombaugh TN, McIntyre NJ. The mini-mental state examination: a comprehensive review. J Am Geriatr Soc. 1992;40:922–35. . 1512391 10.1111/j.1532-5415.1992.tb01992.x

[24] Woodford HJ, George J. Cognitive assessment in the elderly: a review of clinical methods. QJM. 2007;100:469–84. doi: 10.1093/qjmed/hcm051 . 17566006 10.1093/qjmed/hcm051

[25] O'Donnell M, Teo K, Gao P, Anderson C, Sleight P, Dans A, et al Cognitive impairment and risk of cardiovascular events and mortality. Eur Heart J. 2012;33:1777–86. doi: 10.1093/eurheartj/ehs053 . 22551598 10.1093/eurheartj/ehs053

[26] Ekman P, Friesen WV. Pictures of facial affect. Palo Alto, CA, USA: Consulting Psychologists Press, 1976.

[27] Young AW, Rowland D, Calder AJ, Etcoff NL, Seth A, Perrett DI. Facial expression megamix: tests of dimensional and category accounts of emotion recognition. Cognition. 1997;63:271–313. . 9265872 10.1016/s0010-0277(97)00003-6

[28] Harmer CJ, O'Sullivan U, Favaron E, Massey-Chase R, Ayres R, Reinecke A, et al Effect of acute antidepressant administration on negative affective bias in depressed patients. Am J Psychiatry. 2009;166:1178–84. doi: 10.1176/appi.ajp.2009.09020149 . 19755572 10.1176/appi.ajp.2009.09020149

[29] Lewis G, Pelosi AJ, Araya R, Dunn G. Measuring psychiatric disorder in the community: a standardized assessment for use by lay interviewers. Psychol Med. 1992;22:465–86. . 1615114 10.1017/s0033291700030415

[30] Lewis G, Pelosi AJ, Glover E, Wilkinson G, Stansfeld SA, Williams P, et al The development of a computerized assessment for minor psychiatric disorder. Psychol Med. 1988;18:737–45. . 3054992 10.1017/s0033291700008448

[31] Phillips LH, Channon S, Tunstall M, Hedenstrom A, Lyons K. The role of working memory in decoding emotions. Emotion. 2008;8:184–91. doi: 10.1037/1528-3542.8.2.184 . 18410192 10.1037/1528-3542.8.2.184

[32] Henry JD, Phillips LH, Beatty WW, McDonald S, Longley WA, Joscelyne A, et al Evidence for deficits in facial affect recognition and theory of mind in multiple sclerosis. J Int Neuropsychol Soc. 2009;15:277–85. doi: 10.1017/S1355617709090195 . 19203428 10.1017/S1355617709090195

[33] Feinberg TE, Rifkin A, Schaffer C, Walker E. Facial discrimination and emotional recognition in schizophrenia and affective disorders. Arch Gen Psychiatry. 1986;43:276–9. . 3954548 10.1001/archpsyc.1986.01800030094010

[34] Bryson G, Bell M, Lysaker P. Affect recognition in schizophrenia: a function of global impairment or a specific cognitive deficit. Psychiatry Res. 1997;71:105–13. . 9255855 10.1016/s0165-1781(97)00050-4

[35] Yim J, Babbage DR, Zupan B, Neumann D, Willer B. The relationship between facial affect recognition and cognitive functioning after traumatic brain injury. Brain Inj. 2013;27:1155–61. doi: 10.3109/02699052.2013.804203 . 23895556 10.3109/02699052.2013.804203

[36] Peretti CS, Ferreri F, Blanchard F, Bakchine S, Peretti CR, Dobrescu A, et al Normal and pathological aging of attention in presymptomatic Huntington's, Huntington's and Alzheimer's Disease, and nondemented elderly subjects. Psychother Psychosom. 2008;77:139–46. doi: 10.1159/000116607 . 18277060 10.1159/000116607

[37] Reed AE, Carstensen LL. The theory behind the age-related positivity effect. Frontiers in psychology. 2012;3:339 doi: 10.3389/fpsyg.2012.00339 . 23060825 10.3389/fpsyg.2012.00339 PMC3459016

[38] Grau-Olivares M, Arboix A. Mild cognitive impairment in stroke patients with ischemic cerebral small-vessel disease: a forerunner of vascular dementia? Exp Rev Neurother. 2009;9:1201–17. doi: 10.1586/ern.09.73 . 19673608 10.1586/ern.09.73

[39] Fujie S, Namiki C, Nishi H, Yamada M, Miyata J, Sakata D, et al The role of the uncinate fasciculus in memory and emotional recognition in amnestic mild cognitive impairment. Dement Geriatr Cognit Disord. 2008;26:432–9. doi: 10.1159/000165381 . 18957848 10.1159/000165381

[40] Joshi A, Barsuglia JP, Mather MJ, Jimenez EE, Shapira J, Mendez MF. Evaluation of emotional blunting in behavioral variant frontotemporal dementia compared to Alzheimer's disease. Dement Geriatr Cognit Disord. 2014;38:79–88. doi: 10.1159/000357838 . 24603498 10.1159/000357838 PMC4104135

[41] Grainger SA, Henry JD, Phillips LH, Vanman EJ, Allen R. Age deficits in facial affect recognition: The influence of dynamic cues. J Gerontol B Psychol Sci Soc Sci. 2017;72:622–632. doi: 10.1093/geronb/gbv100 . 26530079 10.1093/geronb/gbv100

[42] Kordsachia CC, Labuschagne I, Stout JC. Beyond emotion recognition deficits: A theory guided analysis of emotion processing in Huntington's disease. Neurosci Biobehav Rev. 2017;73:276–92. doi: 10.1016/j.neubiorev.2016.11.020 . 27913281 10.1016/j.neubiorev.2016.11.020

[43] Scholl BJ, Leslie AM. Modularity, development and 'Theory of Mind'. Mind Language. 1999;14:131–153.

[44] Gallese V, Keysers C, Rizzolatti G. A unifying view of the basis of social cognition. Trends Cogn Sci. 2004;8:396–403. doi: 10.1016/j.tics.2004.07.002 . 15350240 10.1016/j.tics.2004.07.002

