# Supplementary material for: The level of cognitive function and recognition of emotions in older adults
Source: PLoS One. 2017 Oct 4;12(10):e0185513. doi: 10.1371/journal.pone.0185513 (PMC5627907; doi:10.1371/journal.pone.0185513)
Supplement: S1 Table — (DOCX) [file pone.0185513.s001.docx]

**S1 Table.** Association between the Mini-Mental State Examination (MMSE) cognitive function test score and accuracy of emotion recognition in the Facial Expression Recognition Task (FERT)

|  | **Anger** | | | **Fear** | | | | **Disgust** | | | **Sadness** | | | | **Happiness** | | | | **Neutral** | | |
| --- | --- | --- | --- | --- | --- | --- | --- | --- | --- | --- | --- | --- | --- | --- | --- | --- | --- | --- | --- | --- | --- |
| **MMSE Score** | **Mean (SE)** | **Mean difference (95% CI)** | **χ^2^ (*P*-value)^a^** | **Mean (SE)** | | **Mean difference (95% CI)** | **χ^2^ (*P*-value)^a^** | **Mean (SE)** | **Mean difference (95% CI)** | **χ^2^ (*P*-value)^a^** | **Mean (SE)** | **Mean difference (95% CI)** | | **χ^2^ (*P*-value)^a^** | **Mean (SE)** | | **Mean difference (95% CI)** | **χ^2^ (*P*-value)^a^** | **Mean (SE)** | **Mean difference (95% CI)** | **χ^2^ (*P*-value)^a^** |
|  |  | Model 1^b^ |  |  | Model 1^b^ | |  |  | Model 1^b^ |  |  | | Model 1^b^ |  |  | Model 1^b^ | |  |  | Model 1^b^ |  |
| 30 | 53.5 (0.5) | Reference |  | 60.1 (0.5) | Reference | |  | 56.8 (0.5) | Reference |  | 47.9 (0.6) | | Reference |  | 70.6 (0.4) | Reference | |  | 67.3 (0.7) | Reference |  |
| 29 | 52.5 (0.4) | -0.96 (-2.28 to 0.36) | 2.0 (0.15) | 58.9 (0.4) | -1.24 (-2.50 to 0.02) | | 3.70 (0.054) | 55.5 (0.4) | -1.33 (-2.48 to -0.19) | 5.3 (0.022, 0.15 ) | 46.8 (0.5) | | -1.04 (-2.55 to 0.47) | 1.8 (0.18) | 69.2 (0.4) | -1.41 (-2.50 to -0.32) | | 6.4 (0.011, 0.08) | 67.5 (0.5) | 0.27 (-1.41 to 1.96) | 0.1 (0.75) |
| 28 | 50.7 (0.5) | -2.72 (-4.13 to -1.30) | 14.1 (<0.001) | 57.7 (0.5) | -2.39 (-3.75 to -1.04) | | 11.9 (<0.001) | 54.7 (0.4) | -2.10 (-3.32 to -0.87) | 11.2 (<0.001) | 46.1 (0.6) | | -1.75 (-3.37 to -0.12) | 4.4 (0.035, 0.25) | 69.6 (0.4) | -1.02 (-2.19 to 0.16) | | 2.9 (0.09) | 66.5 (0.6) | -0.78 (-2.60 to 1.03) | 0.7 (0.40) |
| 27 | 48.3 (0.7) | -5.18 (-6.83 to -3.52) | 37.5 (<0.001) | 54.4 (0.6) | -5.66 (-7.25 to -4.08) | | 48.9 (<0.001) | 52.4 (0.6) | -4.40 (-5.84 to -2.97) | 36.2 (<0.001) | 44.3 (0.8) | | -3.52 (-5.42 to -1.62) | 13.2 (<0.001) | 69.1 (0.5) | -1.56 (-2.93 to -0.18) | | 4.9 (0.026, 0.18) | 64.8 (0.8) | -2.43 (-4.55 to -0.31) | 5.0 (0.025, 0.18) |
| 26 | 46.4 (0.9) | -7.07 (-9.20 to -4.93) | 41.9 (<0.001) | 52.1 (0.9) | -8.01 (-10.07 to -5.96) | | 58.6 (<0.001) | 51.0 (0.8) | -5.85 (-7.71 to -4.00) | 38.3 (<0.001) | 42.5 (1.1) | | -5.40 (-7.85 to -2.95) | 18.7 (<0.001) | 68.3 (0.8) | -2.31 (-4.08 to -0.53) | | 6.5 (0.011, 0.08) | 62.9 (1.2) | -4.38 (-7.12 to -1.64) | 9.8 (0.002, 0.014) |
| 25 | 47.0 (1.5) | -6.46 (-9.57 to -3.35) | 16.6 (<0.001) | 51.8 (1.4) | -8.26 (-11.25 to -5.28) | | 29.4 (<0.001) | 51.0 (1.3) | -5.79 (-8.49 to -3.10) | 17.7 (<0.001) | 41.3 (1.7) | | -6.55 (-10.11 to -2.98) | 13.0 (<0.001) | 66.6 (1.2) | -4.05 (-6.63 to -1.48) | | 9.5 ((0.002, 0.014) | 61.9 (1.9) | -5.38 (-9.37 to -1.40) | 7.0 (0.008, 0.06) |
| 24 | 42.8 (2.0) | -10.63 (-14.63 to -6.64) | 27.3 (<0.001) | 45.5 (1.9) | -14.62 (-18.45 to -10.79) | | 56.0 (<0.001) | 45.1 (1.7) | -11.68 (-15.14 to -8.22) | 43.8 (<0.001) | 35.6 (2.2) | | -12.22 (-16.79 to -7.65) | 27.4 (<0.001) | 65.5 (1.6) | -5.16 (-8.47 to -1.85) | | 9.4 (0.002, 0.014) | 65.9 (2.5) | -1.40 (-6.51 to 3.71) | 0.3 (0.59) |
| <24 | 38.1 (1.8) | -15.32 (-19.1 to -11.5) | 63.3 (<0.001) | 43.6 (1.8) | -16.45 (-20.08 to -12.83) | | 79.3 (<0.001) | 37.4 (1.6) | -19.47 (-22.74 to -16.20) | 136.0 (<0.001) | 36.2 (2.1) | | -11.62 (-15.95 to -7.30) | 27.7 (<0.001) | 64.3 (1.5) | -6.34 (-9.47 to -3.21) | | 15.8 (<0.001) | 51.6 (2.4) | -15.61 (-20.44 to -10.77) | 40.0 (<0.001) |
|  |  | Model 2^c^ |  |  | Model 2^c^ | |  |  | Model 2^c^ |  |  | | Model 2^c^ |  |  | Model 2^c^ | |  |  | Model 2^c^ |  |
| 30 | 54.3 (0.8) | Reference |  | 60.8 (0.8) | Reference | |  | 56.6 (0.7) | Reference |  | 48.2 (0.9) | | Reference |  | 70.5 (0.7) | Reference | |  | 68.5 (1.1) | Reference |  |
| 29 | 53.5 (0.8) | -0.82 (-2.14 to 0.49) | 1.5 (0.22) | 59.7 (0.7) | -1.18 (-2.44 to 0.08) | | 3.4 (0.07) | 55.3 (0.7) | -1.30 (-2.43 to -0.16) | 5.0 (0.026, 0.18) | 47.3 (0.9) | | -0.93 (-2.43 to 0.58) | 1.5 (0.23) | 69.2 (0.6) | -1.31 (-2.40 to -0.22) | | 5.6 (0.018, 0.13) | 68.6 (1.0) | 0.16 (-1.52 to 1.84) | 0.0 (0.86) |
| 28 | 51.7 (0.8) | -2.59 (-4.00 to -1.17) | 12.9 (<0.001) | 58.5 (0.8) | -2.36 (-3.72 to -1.01) | | 11.7 (<0.001) | 54.6 (0.7) | -2.02 (-3.25 to -0.80) | 10.5 (0.001, 0.007) | 46.6 (0.9) | | -1.60 (-3.22 to 0.02) | 3.8 (0.05) | 69.6 (0.7) | -0.90 (-2.07 to 0.27) | | 2.3 (0.13) | 67.5 (1.0) | -0.99 (-2.80 to 0.82) | 1.2 (0.28) |
| 27 | 49.3 (0.9) | -5.01 (-6.66 to -3.35) | 35.2 (<0.001) | 55.4 (0.9) | -5.45 (-7.04 to -3.87) | | 45.4 (<0.001) | 52.4 (0.8) | -4.18 (-5.61 to -2.74) | 32.6 (<0.001) | 44.8 (1.0) | | -3.43 (-5.33 to -1.54) | 12.6 (<0.001) | 69.1 (0.7) | -1.44 (-2.81 to -0.07) | | 4.2 (0.039, 0.27) | 66.1 (1.1) | -2.41 (-4.53 to -0.30) | 5.0 (0.025, 0.18) |
| 26 | 47.5 (1.1) | -6.74 (-8.87 to -4.60 | 38.2 (<0.001) | 53.2 (1.1) | -7.64 (-9.69 to -5.59) | | 53.4 (<0.001) | 51.1 (1.0) | -5.47 (-7.32 to -3.62) | 33.6 (<0.001) | 43.0 (1.3) | | -5.23 (-7.68 to -2.78) | 17.5 (<0.001) | 68.4 (0.9) | -2.14 (-3.91 to -0.37) | | 5.6 (0.018, 0.13) | 64.0 (1.5) | -4.43 (-7.16 to -1.70) | 10.1 (0.002, 0.014) |
| 25 | 48.0 (1.6) | -6.29 (-9.40 to -3.19) | 15.8 (<0.001) | 52.9 (1.6) | -7.92 (-10.89 to -4.94) | | 27.2 (<0.001) | 51.1 (1.4) | -5.44 (-8.13 to -2.76) | 15.8 (<0.001) | 41.7 (1.9) | | -6.51 (-10.07 to -2.96) | 12.9 (<0.001) | 66.5 (1.3) | -4.02 (-6.60 to -1.45) | | 9.4 (0.002, 0.014) | 63.3 (2.1) | -5.19 (-9.16 to -1.22) | 6.6 (0.010, 0.07) |
| 24 | 43.5 (2.1) | -10.77 (-14.75 to -6.79) | 28.2 (<0.001) | 46.4 (2.0) | -14.41 (-18.23 to -10.59) | | 54.8 (<0.001) | 45.1 (1.8) | -11.47 (-14.91 to -8.02) | 42.6 (<0.001) | 35.7 (2.4) | | -12.51 (-17.07 to -7.95) | 28.9 (<0.001) | 65.2 (1.7) | -5.34 (-8.64 to -2.04) | | 10.1 (0.002, 0.014) | 67.3 (2.6) | -1.13 (-6.22 to 3.95) | 0.2 (0.66) |
| <24 | 39.4 (1.9) | -14.91 (-18.69 to -11.12) | 59.7 (<0.001) | 45.3 (1.9) | -15.54 (-19.17 to -11.91) | | 70.4 (<0.001) | 38.1 (1.7) | -18.51 (-21.78 to -15.23) | 122.5 (<0.001) | 36.6 (2.2) | | -11.61 (-15.95 to -7.27) | 27.5 (<0.001) | 64.3 (1.6) | -6.27 (-9.41 to -3.13) | | 15.3 (<0.001) | 53.5 (2.5) | -15.02 (-19.86 to -10.18) | 37.0 (<0.001) |

^a^Second P-value in parentheses indicates the Bonferroni-corrected value.

^b^Model 1 is adjusted for age.

^c^Model 2 is adjusted for age, sex, educational level, depressive symptoms, and antidepressant use.
