# Supplementary material for: The level of cognitive function and recognition of emotions in older adults
Source: PLoS One. 2017 Oct 4;12(10):e0185513. doi: 10.1371/journal.pone.0185513 (PMC5627907; doi:10.1371/journal.pone.0185513)
Supplement: S2 Table — (DOCX) [file pone.0185513.s002.docx]

**S2 Table.** Association between the Mini-Mental State Examination (MMSE) cognitive function test score and misclassification of emotion recognition in the Facial Expression Recognition Task (FERT)

|  | **Anger** | | | **Fear** | | | | **Disgust** | | | **Sadness** | | | | **Happiness** | | | | **Neutral** | | |
| --- | --- | --- | --- | --- | --- | --- | --- | --- | --- | --- | --- | --- | --- | --- | --- | --- | --- | --- | --- | --- | --- |
| **MMSE Score** | **Mean (SE)** | **Mean difference (95% CI)** | **χ^2^ (*P*-value)^a^** | **Mean (SE)** | | **Mean difference (95% CI)** | **χ^2^ (*P*-value)^a^** | **Mean (SE)** | **Mean difference (95% CI)** | **χ^2^ (*P*-value)^a^** | **Mean (SE)** | **Mean difference (95% CI)** | | **χ^2^ (*P*-value)^a^** | **Mean (SE)** | | **Mean difference (95% CI)** | **χ^2^ (*P*-value)^a^** | **Mean (SE)** | **Mean difference (95% CI)** | **χ^2^ (*P*-value)^a^** |
|  |  | Model 1^b^ |  |  | Model 1^b^ | |  |  | Model 1^b^ |  |  | | Model 1^b^ |  |  | Model 1^b^ | |  |  | Model 1^b^ |  |
| 30 | 3.5 (0.1) | Reference |  | 5.6 (0.1) | Reference | |  | 3.7 (0.1) | Reference |  | 5.2 (0.1) | | Reference |  | 2.9 (0.1) | Reference | |  | 26.2 (0.3) | Reference |  |
| 29 | 3.8 (0.1) | 0.25 (-0.05 to 0.56) | 2.7 (0.10) | 5.6 (0.1) | -0.01 (-0.37 to 0.36) | | 0.0 (0.97) | 4.1 (0.1) | 0.38 (0.05 to 0.72) | 5.1 (0.024, 0.17) | 5.3 (0.1) | | 0.10 (-0.27 to 0.48) | 0.3 (0.59) | 2.8 (0.1) | -0.09 (-0.40 to 0.22) | | 0.3 (0.57) | 26.7 (0.2) | 0.50 (-0.27 to 1.27) | 1.6 (0.20) |
| 28 | 3.7 (0.1) | 0.20 (-0.13 to 0.52) | 1.4 (0.23) | 5.8 (0.1) | 0.19 (-0.20 to 0.58) | | 0.9 (0.35) | 4.5 (0.1) | 0.79 (0.43 to 1.15) | 18.6 (<0.001) | 5.6 (0.1) | | 0.40 (-0.00 to 0.80) | 3.8 (0.05) | 3.2 (0.1) | 0.28 (-0.05 to 0.61) | | 2.7 (0.10) | 26.6 (0.3) | 0.40 (-0.42 to 1.23) | 0.9 (0.34) |
| 27 | 4.1 (0.2) | 0.63 (0.25 to 1.01) | 10.7 (0.001, 0.007) | 6.3 (0.2) | 0.71 (0.25 to 1.17) | | 9.2 (0.003, 0.021) | 5.2 (0.2) | 1.45 (1.03 to 1.87) | 45.9 (<0.001) | 6.2 (0.2) | | 1.07 (0.59 to 1.54) | 19.6 (<0.001) | 3.2 (0.2) | 0.35 (-0.03 to 0.74) | | 3.2 (0.07) | 26.8 (0.4) | 0.57 (-0.39 to 1.54) | 1.4 (0.24) |
| 26 | 5.0 (0.2) | 1.50 (1.01 to 1.99) | 36.2 (<0.001) | 6.4 (0.3) | 0.80 (0.21 to 1.40) | | 7.0 (0.008, 0.06) | 5.5 (0.2) | 1.78 (1.23 to 2.32) | 41.1 (<0.001) | 6.2 (0.3) | | 1.09 (0.48 to 1.69) | 12.2 (<0.001) | 3.9 (0.2) | 1.04 (0.54 to 1.54) | | 16.8 (<0.001) | 26.9 (0.6) | 0.64 (-0.61 to 1.88) | 1.0 (0.32) |
| 25 | 5.1 (0.3) | 1.60 (0.89 to 2.32) | 19.5 (<0.001) | 6.9 (0.4) | 1.32 (0.46 to 2.19) | | 9.0 (0.003, 0.021) | 6.4 (0.4) | 2.69 (1.90 to 3.48) | 44.6 (<0.001) | 6.5 (0.4) | | 1.39 (0.51 to 2.28) | 9.5 (0.002, 0.014) | 3.6 (0.3) | 0.76 (0.03 to 1.49) | | 4.2 (0.040, 0.28) | 25.9 (0.9) | -0.32 (-2.13 to 1.49) | 0.1 (0.73) |
| 24 | 5.6 (0.4) | 2.08 (1.17 to 3.00) | 20.0 (<0.001) | 7.1 (0.5) | 1.44 (0.33 to 2.55) | | 6.5 (0.011, 0.08) | 6.6 (0.5) | 2.92 (1.91 to 3.94) | 32.0 (<0.001) | 6.6 (0.6) | | 1.42 (0.28 to 2.56) | 6.0 (0.015, 0.11) | 4.2 (0.5) | 1.33 (0.40 to 2.26) | | 7.8 (0.005, 0.035) | 28.9 (1.1) | 2.69 (0.37 to 5.02) | 5.2 (0.023, 0.16) |
| <24 | 7.0 (0.4) | 3.47 (2.60 to 4.33) | 61.8 (<0.001) | 9.8 (0.5) | 4.14 (3.09 to 5.19) | | 59.8 (<0.001) | 8.6 (0.5) | 4.91 (3.95 to 5.87) | 100.6 (<0.001) | 9.2 (0.5) | | 4.03 (2.95 to 5.11) | 53.9 (<0.001) | 6.3 (0.4) | 3.45 (2.57 to 4.33) | | 59.0 (<0.001) | 22.5 (1.1) | -3.71 (-5.91 to -1.51) | 10.9 (<0.001) |
|  |  | Model 2^c^ |  |  | Model 2^c^ | |  |  | Model 2^c^ |  |  | | Model 2^c^ |  |  | Model 2^c^ | |  |  | Model 2^c^ |  |
| 30 | 3.7 (0.2) | Reference |  | 5.8 (0.2) | Reference | |  | 3.8 (0.2) | Reference |  | 4.7 (0.2) | | Reference |  | 2.8 (0.2) | Reference | |  | 25.7 (0.5) | Reference |  |
| 29 | 4.0 (0.2) | 0.28 (-0.02 to 0.58) | 3.2 (0.07) | 5.9 (0.2) | 0.01 (-0.35 to 0.38) | | 0.0 (0.95) | 4.2 (0.2) | 0.37 (0.04 to 0.70) | 4.7 (0.030, 0.21) | 4.8 (0.2) | | 0.10 (-0.27 to 0.48) | 0.3 (0.58) | 2.7 (0.2) | -0.09 (-0.40 to 0.22) | | 0.4 (0.56) | 26.1 (0.4) | 0.39 (-0.37 to 1.15) | 1.0 (0.31) |
| 28 | 3.9 (0.2) | 0.22 (-0.11 to 0.54) | 1.7 (0.19) | 6.0 (0.2) | 0.21 (-0.18 to 0.60) | | 1.1 (0.30) | 4.6 (0.2) | 0.78 (0.42 to 1.14) | 18.1 (<0.001) | 5.1 (0.2) | | 0.42 (0.02 to 0.82) | 4.2 (0.041, 0.29) | 3.1 (0.2) | 0.28 (-0.05 to 0.61) | | 2.8 (0.09) | 26.0 (0.5) | 0.27 (-0.55 to 1.09) | 0.4 (0.52) |
| 27 | 4.3 (0.2) | 0.60 (0.23 to 0.98) | 9.8 (0.002, 0.014) | 6.5 (0.2) | 0.69 (0.23 to 1.15) | | 8.5 (0.004, 0.028) | 5.2 (0.2) | 1.39 (0.97 to 1.81) | 42.1 (<0.001) | 5.8 (0.3) | | 1.06 (0.59 to 1.53) | 19.4 (<0.001) | 3.1 (0.2) | 0.34 (-0.04 to 0.73) | | 3.0 (0.08) | 26.3 (0.5) | 0.53 (-0.42 to 1.49) | 1.2 (0.28) |
| 26 | 5.2 (0.3) | 1.47 (0.99 to 1.96) | 35.0 (<0.001) | 6.6 (0.3) | 0.79 (0.19 to 1.38) | | 6.7 (0.010, 0.07) | 5.5 (0.3) | 1.69 (1.15 to 2.23) | 37.1 (<0.001) | 5.8 (0.3) | | 1.07 (0.46 to 1.68) | 11.9 (<0.001) | 3.8 (0.3) | 1.02 (0.52 to 1.52) | | 15.9 (<0.001) | 26.3 (0.7) | 0.52 (-0.72 to 1.75) | 0.7 (0.41) |
| 25 | 5.2 (0.4) | 1.55 (0.84 to 2.26) | 18.3 (<0.001) | 7.1 (0.5) | 1.29 (0.42 to 2.15) | | 8.5 (0.004, 0.028) | 6.5 (0.4) | 2.63 (1.84 to 3.42) | 42.7 (<0.001) | 6.1 (0.5) | | 1.35 (0.47 to 2.24) | 9.0 (0.003, 0.021) | 3.6 (0.4) | 0.75 (0.02 to 1.47) | | 4.1 (0.044, 0.31) | 25.4 (0.9) | -0.32 (-2.12 to 1.48) | 0.12 (0.73) |
| 24 | 5.7 (0.5) | 2.01 (1.10 to 2.92) | 18.7 (<0.001) | 7.2 (0.6) | 1.39 (0.28 to 2.50) | | 6.0 (0.014), 0.10) | 6.7 (0.5) | 2.90 (1.89 to 3.91) | 31.6 (<0.001) | 6.1 (0.6) | | 1.38 (0.25 to 2.52) | 5.7 (0.017, 0.12) | 4.1 (0.5) | 1.31 (0.38 to 2.24) | | 7.6 (0.006, 0.042) | 28.6 (1.2) | 2.91 (0.60 to 5.21) | 6.1 (0.014, 0.10) |
| <24 | 7.0 (0.4) | 3.27 (2.40 to 4.13) | 54.8 (<0.001) | 9.8 (0.5) | 3.98 (2.93 to 5.04) | | 54.9 (<0.001) | 8.5 (0.5) | 4.68 (3.72 to 5.64) | 90.8 (<0.001) | 8.7 (0.6) | | 3.96 (2.88 to 5.04) | 51.7 (<0.001) | 6.2 (0.5) | 3.38 (2.49 to 4.26) | | 56.0 (<0.001) | 22.2 (1.1) | -3.54 (-5.74 to -1.35) | 10.0 (0.002, 0.014) |

^a^Second *P*-value in parentheses indicates the Bonferroni-corrected value.

^b^Model 1 is adjusted for age.

^c^Model 2 is adjusted for age, sex, educational level, depressive symptoms, and antidepressant use.
