# Supplementary material for: The level of cognitive function and recognition of emotions in older adults
Source: PLoS One. 2017 Oct 4;12(10):e0185513. doi: 10.1371/journal.pone.0185513 (PMC5627907; doi:10.1371/journal.pone.0185513)
Supplement: S3 Table — (DOCX) [file pone.0185513.s003.docx]

**S3 Table.** Bivariate Pearson correlations (r) between Mini Mental State Examination (MMSE) total score and subscores and accuracy of emotion recognition in the Facial Expression Recognition Task (FERT)

|  | FERT accuracy score | | | | | |
| --- | --- | --- | --- | --- | --- | --- |
| MMSE score | Anger  r | Fear  r | Disgust  r | Sadness  r | Happiness  r | Neutral  r |
| Total score | 0.22^c^ | 0.26^c^ | 0.25^c^ | 0.17^c^ | 0.10^c^ | 0.13^c^ |
| Attention/concentration | 0.09^c^ | 0.12^c^ | 0.14^c^ | 0.08^c^ | 0.04^a^ | 0.07^c^ |
| Language skills | 0.17^c^ | 0.18^c^ | 0.16^c^ | 0.13^c^ | 0.09^c^ | 0.10^c^ |
| Memory recall | 0.15^c^ | 0.18^c^ | 0.18^c^ | 0.12^c^ | 0.06^c^ | 0.06^c^ |
| Orientation | 0.13^c^ | 0.15^c^ | 0.13^c^ | 0.08^c^ | 0.04^b^ | 0.08^c^ |

^a^*P*<0.05

^b^*P*<0.01

^c^*P*<0.001
